# The relative contribution of individual quality and changing climate as drivers of lifetime reproductive success in a short-lived avian species

**DOI:** 10.1038/s41598-020-75557-w

**Published:** 2020-11-13

**Authors:** Lisha L. Berzins, Russell D. Dawson, Christy A. Morrissey, Robert G. Clark

**Affiliations:** 1 Department of Biology, University of Saskatchewan, Saskatoon, SK S7N 5E2 Canada; 2 Ecosystem Science and Management Program, University of Northern British Columbia, Prince George, BC V2N 4Z9 Canada; 3 School of Environment and Sustainability, University of Saskatchewan, Saskatoon, SK S7N 5C8 Canada; 4 Environment and Climate Change Canada, Saskatoon, SK S7N 0X4 Canada

**Keywords:** Ecology, Climate-change ecology, Conservation biology, Population dynamics

## Abstract

Animal populations are influenced strongly by fluctuations in weather conditions, but long-term fitness costs are rarely explored, especially in short-lived avian species. We evaluated the relative contributions of individual characteristics and environmental conditions to lifetime reproductive success (LRS) of female tree swallows (*Tachycineta bicolor*) from two populations breeding in contrasting environments and geographies, Saskatchewan and British Columbia, Canada. Female swallows achieved higher LRS by breeding early in the season and producing more fledglings. Other measures of female quality had virtually no influence on LRS. Genetic factors did not predict LRS, as there was no correlation between life-history components for sister pairs nor between mothers and their daughters. Instead, climate variability—indexed by spring pond density (i.e., abundance of wetland basins holding water) during years when females bred—had strong positive effects on female LRS in more arid Saskatchewan but only weak positive effects of moisture conditions were detected in wetter British Columbia. Overall, several life history trait correlates of LRS were similar between populations, but local environmental factors experienced by individuals while breeding produced large differences in LRS. Consequently, variable and extreme environmental conditions associated with changing climate are predicted to influence individual fitness of distinct populations within a species’ range.

## Introduction

Fluctuations in weather conditions play a major role in dynamics of animal populations by affecting survival and reproduction^1^, and these effects may be particularly pronounced in years with extreme weather events^2^. For instance, severe heat, cold or drought can reduce survival^3,4^ and result in reproductive failure^5^. The frequency and severity of extremes in weather conditions are predicted to increase in the future^6^, with heavy precipitation, warmer temperatures, extreme floods and drought projected for the northern hemisphere^7^. Studies assessing the effects of weather variability generally focus on short-term effects on survival and reproduction, whereas the effects on the long-term fitness of animals remain underexplored^8^. One approach to evaluating how weather variability and climate extremes influence avian fitness is to determine their individual and combined effects on the total numbers of offspring produced by individuals over their lifetime that contribute to future generations, i.e., lifetime reproductive success (LRS)^9,10^. A better understanding of the major drivers affecting LRS is urgently needed to predict population responses to a rapidly changing environment.

Decades of research across a variety of taxa have demonstrated large variation among individuals in reproductive performance, with relatively few individuals producing most of the surviving offspring^9,10^. Local weather conditions vary geographically, influencing the production of offspring by some individuals^11^, and emphasizing the importance of evaluating populations across contrasting environments. Alternatively, the success of individuals at producing offspring that contribute to future generations compared to those producing few to no offspring in their lifetime may also reflect underlying differences in individual quality^12–14^. For example, some individuals in a population may be consistently successful at producing young, even in the face of contrasting environmental conditions^15^. Indeed, some studies have demonstrated that individual traits reflecting quality, such as body size^16^, timing of breeding^17,18^, age of first reproduction^19^, and longevity^20,21^ are positively related to LRS. The relative contribution of these individual characteristics to variation in LRS is nonetheless often weak, suggesting variation in LRS may instead be driven by stochastic processes^22,23^.

Ultimately, there are still unresolved debates on whether the traits of individuals or environmental stochasticity are the most important drivers of variation in LRS^14,23,24^. Part of the uncertainty may arise from the context of events. For example, in southern fulmars (*Fulmarus glacialoides*), when conditions were favorable, environmental stochasticity influenced LRS, but during poor conditions, heterogeneity in individual traits explained greater variation in LRS^25^. Additionally, the majority of studies focus on long-lived species, whereas short-lived species that breed only once or twice in their lifetime may be less able to contend with fluctuating or extreme environmental conditions. Lastly, measurements of LRS, at least in avian studies, commonly assume that the “number of fledglings” (survive to fledging age) is a reliable approximation of fitness instead of more accurately determining the “number of recruits” (survive to breeding age), which may obscure the interpretation of LRS drivers^16,20^.

Here, we use confirmatory path analysis^26^ to simultaneously evaluate the relative importance of individual characteristics (i.e., age of first breeding, body condition, timing of breeding, and number of breeding attempts [longevity]) and environmental factors (i.e., number of wetland basins containing water [pond abundance], soil moisture conditions, and local cold weather severity) for LRS of adult female tree swallows (*Tachycineta bicolor*). While path analysis has been performed in relatively long-lived species^20,27^, this approach has not been used to disentangle whether individual traits or environmental factors are the dominant drivers of LRS in short-lived species. Apparent LRS, the number of recruited offspring to the study site, was calculated for large samples of known-age adult female tree swallows based on > 15 year longitudinal data sets from two geographically distinct populations in western Canada (Saskatchewan [SK] and British Columbia [BC]; > 1200 km apart) with distinct local environmental conditions (Table S1). The SK site in the prairies is more arid and experiences continental climate extremes driving fluctuations in pond abundances during drought-deluge cycles^28^, whereas the BC site has higher moisture and experiences unpredictable, severe cold and wet weather events, particularly early in the breeding season^29^. Under projected climate change scenarios, the frequency of these extreme events is predicted to increase^6^, warranting greater understanding of their effects. Tree swallows feed primarily on aerial insects that emerge from aquatic environments during warmer weather^30^ but are negatively affected by cold snaps^31^. We hypothesized that in years with more abundant ponds or warmer and wetter soil moisture conditions present when females bred would reflect increased food supply^32^ or higher quality food, resulting in the production of larger broods^33^. We also examined potential heritability of life-history components by assessing correlations in lifetime number of breeding attempts, eggs laid, fledglings produced, and timing of breeding for sister pairs and between mothers and their daughters.

## Results

### General patterns of reproduction and apparent LRS of known-age adult females

**Figure 1:**
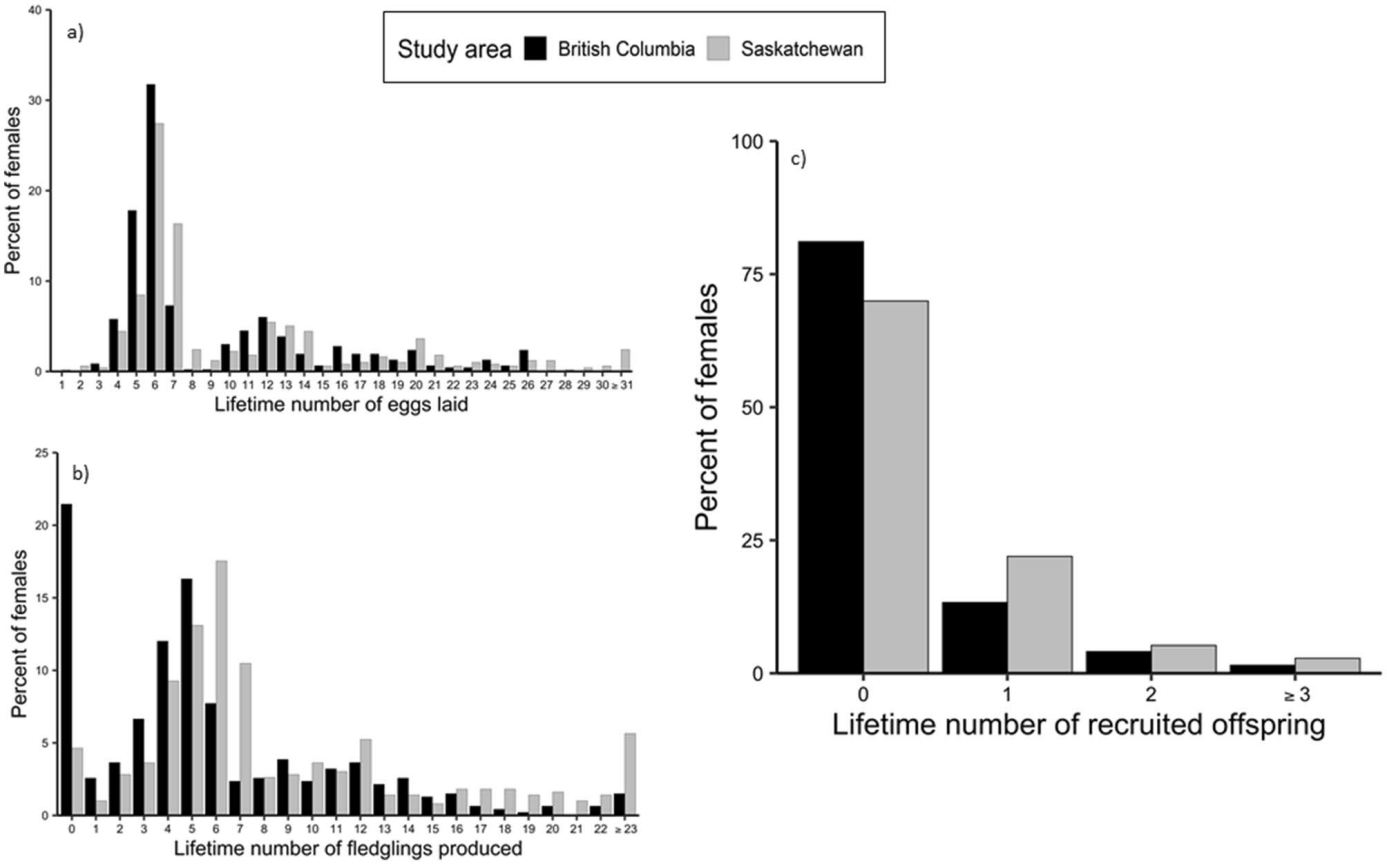
Lifetime numbers of (**a**) total eggs laid, (**b**) fledged nestlings and (**c**) recruited offspring produced by known-age adult female tree swallows in Saskatchewan (1990–2015; *n* = 496) and British Columbia (2001–2015; *n* = 466).

In SK, most female tree swallows were first detected breeding at either 1 (48%) or 2 (37%) years old; few were ≥ 3 (15%). In BC, most females were detected breeding for the first time as yearlings (90%) while the remaining females (10%) bred at ≥ 2 years old. In SK, 60% of female tree swallows made a single breeding attempt in their lifetime, while 21% bred twice, 10% bred three times, and fewer than 8% bred ≥ 4 times. The number of breeding attempts by females in BC was similar: 63%, 20%, 11% and 5% corresponding to 1, 2, 3, and ≥ 4 attempts, respectively. In both study areas, > 50% of adult females laid 5–7 eggs (Fig. 1a), and 50% and 35%, SK and BC respectively, fledged between 4–7 nestlings in their lifetime (Fig. 1b), reflecting the average for a single breeding attempt made by most females. The number of adult females failing to produce any fledged offspring differed by site: in SK, < 5% of females failed to fledge offspring, whereas 21% of females in BC produced no fledglings (Fig. 1b).

Apparent LRS varied widely among adult females at both study sites (Fig. 1c); the mean (± SE) number of recruits produced by females was 0.42 ± 0.04 at SK (range: 0 to 5) and 0.26 ± 0.03 at BC (range: 0 to 4). Low LRS of females suggests that the majority, 70% in SK and 81% in BC, produced no locally recruited offspring, whereas the remaining 30% of females in SK produced 1.41 ± 0.06 recruits and 19% in BC produced 1.40 ± 0.07 recruits.

### Saskatchewan LRS path model

**Figure 2:**
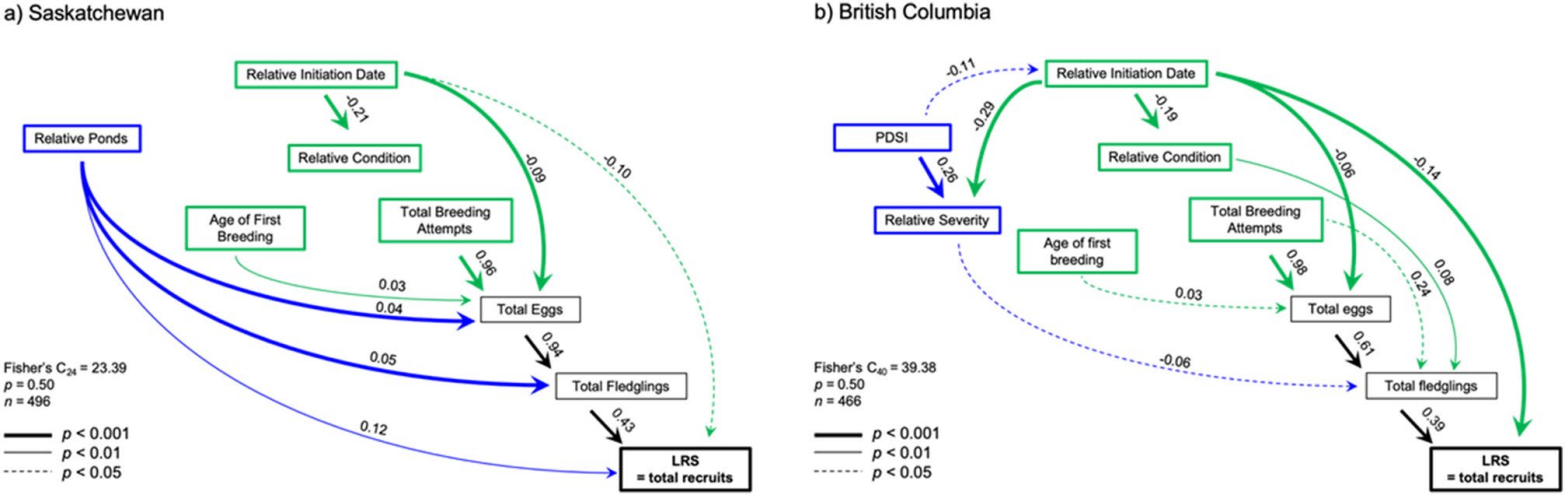
Path diagram summarizing direct and indirect effects of explanatory variables on lifetime reproductive success of known-age adult female tree swallows breeding in (**a**) Saskatchewan and (**b**) British Columbia. Line thickness indicates the relative strength of the path. See “Materials and methods” for description of calculation of explanatory variables. Green represents individual characteristics of females, whereas blue represents environmental factors. Age of first breeding for females in Saskatchewan was 1 year old for 48%, 2 years old for 37%, and ≥ 3 years old for 15%, whereas in British Columbia, 90% of females bred at 1 year old and the other 10% bred at ≥ 2 years old.

The original hypothesized path model for SK tree swallows had a poor fit to the data (Fisher’s C_28_ = 54.49, *p* = 0.002, *n* = 496). Two significant pathways were added (Relative Ponds → Total Eggs and Relative Ponds → LRS), and five non-significant pathways were removed (Total Breeding Attempts → LRS; Relative Initiation Date → Total Breeding Attempts; Relative Condition → Total Breeding Attempts; Age of First Breeding → Total Breeding Attempts; and Relative Ponds → Relative Initiation Date). Overall, the final path model had a good fit to the data (Fisher’s C_24_ = 23.39, *p* = 0.50, Fig. 2a).

The final path model revealed that the lifetime number of fledglings (Total Fledglings) had the strongest direct positive effect on apparent LRS (Fig. 2a). Lifetime number of eggs laid (Total Eggs) and Total Breeding Attempts (adult female survival) also had strong positive effects on LRS indirectly through lifetime fledgling production (Total Fledglings; Table 1, Fig. 2a). Lifetime clutch initiation date of females (Relative Initiation Date) had a direct negative effect on apparent LRS (Table 1, Fig. 2a) and was further strengthened by an indirect negative effect of lifetime clutch initiation date (Relative Initiation Date) on lifetime egg production (Total Eggs) (Table 1). Lastly, the abundance of ponds present in the landscape in the year(s) females bred (Relative Ponds) had a direct positive effect on LRS (Table 1, Fig. 2a). This effect was strengthened by two indirect pathways from Relative Ponds → Total Eggs and Relative Ponds → Total Fledglings, all leading to higher apparent LRS (Table 1). Additionally, the negative effects of breeding later (Relative Initiation Date) on LRS (− 0.01) and egg production (Total Eggs) (− 0.004) appeared to be dampened by pond density (Relative Ponds) because later breeding females tended to lay more eggs and produce more recruits when ponds were more abundant.

**Table 1:** Summary of direct (standardized path coefficients) and total effects of individual traits and environmental factors on the lifetime reproductive success (LRS) of known-age adult female tree swallows breeding in Saskatchewan (1990–2015) and British Columbia (2001–2015).

LRS correlate	Site			
	Saskatchewan		British Columbia	
	Direct	Total	Direct	Total
Total fledglings	0.43	0.43	0.39	0.39
Total eggs	–	0.40	–	0.24
Total breeding attempts	–	0.39	–	0.33
Relative initiation date	− 0.10	− 0.14	− 0.14	− 0.16
Relative condition	–	0.00	–	0.03
Age of first breeding	–	0.01	–	0.006
Relative ponds	0.12	0.16	N/A	N/A
Relative weather severity	N/A	N/A	–	− 0.02
Average PDSI^a^	N/A	N/A	–	0.01

Refer to “Materials and methods” for details about calculation of total effects.

^a^Average PDSI—Mean Palmer drought severity index for June and July averaged over all lifetime breeding attempts (see “Materials and methods” for details).

Reproductive age (Age of First Breeding) of SK females had a relatively weak positive effect on LRS, but only indirectly via lifetime egg production (0.012), suggesting that delayed breeding had little influence on LRS. A strong negative path between lifetime clutch initiation date (Relative Initiation Date) and body condition (Relative Condition) was detected, indicating that late-nesting females were light-weight for their body size (Fig. 2a); however, female body condition had no effect on apparent LRS.

Total number of breeding attempts (Total Breeding Attempts) contributed indirectly to higher LRS (see above), and not directly as hypothesized. None of the individual trait variables we initially hypothesized as important drivers of lifetime fitness (i.e., Relative Condition, Relative Initiation Date and Age of First Breeding) had a significant relationship with overall number of breeding attempts (Total Breeding Attempts) and were not retained in the final LRS model (Fig. 2a).

### British Columbia LRS path model

The original hypothesized path model for BC also had poor fit to the data (Fisher’s C_38_ = 87.27, *p* < 0.0001, *n* = 466). Three significant paths (Average PDSI (soil moisture conditions) → Relative Initiation Date; Relative Condition → Total Fledglings; and Total Breeding Attempts → Total Fledglings) were added to the model. Several non-significant paths (PDSI → Total Fledglings; Age of First Breeding → Total Breeding Attempts; Relative Initiation Date → Total Breeding Attempts; Relative Condition → Total Breeding Attempts; and Total Breeding Attempts → LRS) were removed (all *p* > 0.50). Overall, the final path model had a good fit to the data (Fisher’s C_40_ = 39.38, *p* = 0.50, Fig. 2b).

Lifetime production of fledglings (Total Fledglings) was the strongest direct predictor of female LRS (Table 1). As expected, lifetime number of eggs laid (Total Eggs) and adult female survival (Total Breeding Attempts) were also important contributors to LRS by increasing the total number of fledglings produced by females (Table 1, Fig. 2b). Lifetime clutch initiation date (Relative Initiation Date) had a direct negative effect on LRS (Fig. 2b), and the effect was strengthened by an indirect pathway through lifetime egg production (Total Eggs), suggesting that late breeding females had lower LRS (Table 1, Fig. 2b). Age (Age of First Breeding) and body condition (Relative Condition) of females only weakly influenced LRS through indirect paths. Females that delayed breeding until two or three years old tended to lay more eggs (Fig. 2b), but this had no effect on LRS (Table 1). Females in relatively higher body condition over their lifetime generally produced more fledglings and more recruits (Fig. 2b), although this effect was relatively weak (Table 1).

The number of breeding attempts made by females did not increase apparent LRS directly, as hypothesized (Fig. 2b), although a weak direct path from Total Breeding Attempts → Total Fledglings was detected (Fig. 2b), strengthening the effect of number of breeding attempts on overall LRS (see above). Again, none of the trait variables hypothesized to influence a female’s number of breeding attempts (Total Breeding Attempts) were retained in the final model (Fig. 2b).

Several indirect pathways including soil moisture conditions (Average PDSI) yielded a weak positive effect on LRS (Table 1), suggesting that wetter soil conditions in BC tended to increase LRS (Fig. 2b). Soil moisture had a positive effect on LRS via earlier clutch initiation date (Relative Initiation Date), but this was countered by early breeding females being more likely to experience cold weather (Relative Severity), which had a negative effect on fledgling production (Total Fledglings) (Fig. 2b).

### Heritability of life-history traits: sister and mother-daughter pairs

Across sites, there was no significant correlation in total fledglings, eggs or breeding attempts or relative initiation date between mothers and their daughters (*p*-values > 0.48) nor with sister pairs (*p*-values > 0.29) (Table 2).

**Table 2:** Correlations for life-history components between mothers and their daughters and sister pairs of known-age adult female tree swallows breeding in Saskatchewan (1990–2015) and British Columbia (2001–2015).

LRS component	Mother–daughter			Sister–sister		
	Spearman R	*p*	*n*	Spearman R	*p*	*n*
Total fledglings	0.008	0.93	140	− 0.21	0.29	26
Total eggs	− 0.02	0.80	140	− 0.13	0.52	26
Total breeding attempts	0.06	0.48	140	− 0.09	0.65	26
Relative initiation date	0.06	0.51	140	− 0.007	0.97	26

## Discussion

Our results for LRS in two geographically distinct populations using hundreds of known-age female tree swallows monitored for > 15 years were consistent with four important generalizations: (1) most females are short-lived and breed only once in their lifetime; (2) early annual onset of breeding is key to their long-term reproductive success; (3) age of first breeding, individual quality and genetics were not strong predictors of traits related to LRS; and (4) environmental conditions experienced in the year(s) females bred generated large variation in LRS. These results have important conservation implications as climate change can alter local environmental conditions on the breeding grounds that can directly affect population demographics in passerines with short lifespans. Overall, consistent with other animal models, the dominant drivers of tree swallow LRS are timing of initiating breeding, longevity and environmental conditions that influence production of offspring^17,21,34^. Additionally, higher lifetime production of fledglings was the strongest correlate of female apparent LRS, lending support for the common use and assumption of fledging success as a proxy for LRS in short-lived passerines^35^.

Life-history theory predicts that individuals making greater investment in current reproduction should suffer lower subsequent survival or future reproduction^36^. This fitness trade-off has been observed in LRS studies in some species (e.g., eastern kingbirds (*Tyrannus tyrannus*)^37^, song sparrows (*Melospiza melodia*)^38^, and northern elephant seals (*Mirounga angustirostris*)^21^). In contrast, our results suggest that survival and reproduction may positively covary in tree swallows. We speculated that this pattern is indicative of individual quality where some individuals are better able to invest in self-maintenance and still achieve higher LRS^39^. Interestingly, none of the individual trait characteristics we measured, aside from timing of breeding, were related to longevity or LRS. Although stochasticity can generate a positive correlation between survival and reproduction^22^, our results are consistent with previous experimental research in tree swallows failing to detect direct costs of reproduction^40^. For example, females raising experimentally enlarged broods fledged more offspring with no apparent cost to their return rates or future breeding success^41,42^. Instead, Shutler et al*.*^42^ hypothesized that fitness in tree swallows is constrained more by timing of initiating breeding, since females breeding later in the season produced fewer recruits in their lifetime. This phenomenon was confirmed in our study systems, each with much larger samples of known-age birds.

In seasonal environments, one of the most prominent determinants of reproductive success is timing of initiating breeding^43^. Females that breed late in the season generally lay fewer eggs in their lifetime and have lower apparent LRS^18,20^. That we detected a stronger effect of later-season breeding on LRS than on the lifetime number of eggs supports previous studies demonstrating a benefit of early-season breeding on post-fledging survival^20,44^. The relative number of recruits decreased with lifetime clutch initiation date, suggesting that females who generally breed early in the season produce the majority of recruits, while those that are relatively late-season breeders produce only a few locally recruited offspring (Fig. S1). While we cannot rule out the possibly that offspring hatched later in the season permanently dispersed outside the study sites, previous research in diverse animal species has shown that later hatched (or born) offspring are generally lower quality and have lower apparent return rates than offspring produced early in the season^45,46^.

Declines in reproductive success associated with breeding later in the season are hypothesized to be the consequence of differences in individual quality or environmental conditions between early- and late-season breeders^47^. Females that bred early in the season had higher body condition compared to later-season breeders, consistent with the individual quality hypothesis, yet condition had virtually no effect on LRS. While we recognize that we were unable to control for potential effects on the body condition of females such as time of day that females were captured or weather^48^, links between adult female body condition and annual reproductive success are seldom detected in tree swallows^29,49,50^. Additionally, female body condition was less influential for recruitment or LRS in other species, such as the great tit (*Parus major*)^51^, common pochard (*Aythya ferina*)^52^, and northern wheatear (*Oenanthe oenanthe*)^53^. Instead, our results are consistent with experimental studies showing that the date in the season when a female breeds is causally related to the number of fledging or recruiting offspring^29,54^. Seasonally declining food supply is one mechanism hypothesized to underlie differences in the number of fledglings or recruits produced by early- and late-season breeding females^47,50,55^. While we lack long-term data on aerial insect abundance, Harriman et al*.*^50^ previously showed that insect biomass is generally lower at the BC site, which may explain the higher number of females producing no recruits and the stronger negative effect of timing of breeding on LRS at BC. Additionally, abundant ponds reflecting higher food supply^32^ likely dampened the negative effect of late season breeding on LRS at SK.

Similar with other studies that have attempted to simultanously disentangle effects of individual quality versus environmental factors for offspring recruitment or LRS^20,52^, we found that body condition and reproductive age of females had relatively weak effects on LRS. Although Winkler et al*.*^18^ recently reported that clutch initiation date was correlated for mothers and their daughters that began breeding for the first time as yearlings, we found no significant correlation among mothers and their daughters, even when we considered only those breeding as yearlings (*p* = 0.44, *n* = 50). The lack of correlation between sisters and mother-daughter pairs in clutch initiation date and other life-history components further indicates that genetic variation has little influence on LRS, although we recognize that our sample sizes are small, and some sisters may not share a genetic father due to extra-pair paternity. In other species, age of first breeding had positive effects on LRS, such as goshawks (*Accipiter gentilis*)^19^ and blue-footed boobies (*Sula nebouxii*)^56^; however, benefits of this strategy to LRS may be limited to species with a long lifespan and early costs of reproduction, or when delaying breeding to an older age does not result in lower LRS^57^, as in tree swallows. Overall, our conclusions are similar to work in other species reporting little effect of female traits on LRS^20,58^ and a lack of heritability of LRS^27,59^. This may reflect the larger effect of environmental conditions relative to genetic variation on lifetime fitness of individuals in short-lived species^60,61^.

Environmental factors have been reported as important drivers of variation in LRS among individuals, but these studies are generally limited to a single population^15,62^ or populations within close proximity^58^. Our results show that environmental drivers causing variation in LRS can vary across a species’ range and may explain geographic differences in population changes. In SK, females that bred when ponds were abundant were able to make greater investments in reproduction, suggesting that aquatic insect food supply or quality can offset reproductive costs^32,63^. Although we were unable to directly assess links between pond abundance and survival because our measure of Relative Ponds was standardized to be independent of lifetime breeding attempts, higher apparent survival of females in years following higher pond abundance has been previously reported^64^. Thus, ponds are critically important for terrestrial species that rely on aquatic food resources, highlighting the urgent need to protect prairie wetland basins from further loss and degradation.

At the BC site where wetter environmental conditions contrast those in more arid SK, we observed that wetter soil moisture conditions did not lead to higher LRS, consistent with Harriman’s^33^ report that PDSI was not an important predictor of juvenile apparent survival. PDSI had a weak positive effect on lifetime relative initiation date; however, early-season breeding females were more likely to encounter cold weather while raising nestlings, as reported for an Ontario population of tree swallows^65^. Although unpredictable, cold weather events can have devastating effects on nestling survival^5^, our measure of cold severity did not fully account for the low total number of fledglings produced by some females at BC. Weegman et al*.*^11^ also found that low fledgling success at this BC site was unrelated to local weather. Previous work in tree swallows suggests that the production of fledglings is an important driver of population growth^11,66^, and is the strongest determinant of LRS; therefore, mechanisms underlying lower fledging success at the BC site warrant further investigation.

**Figure 3:**
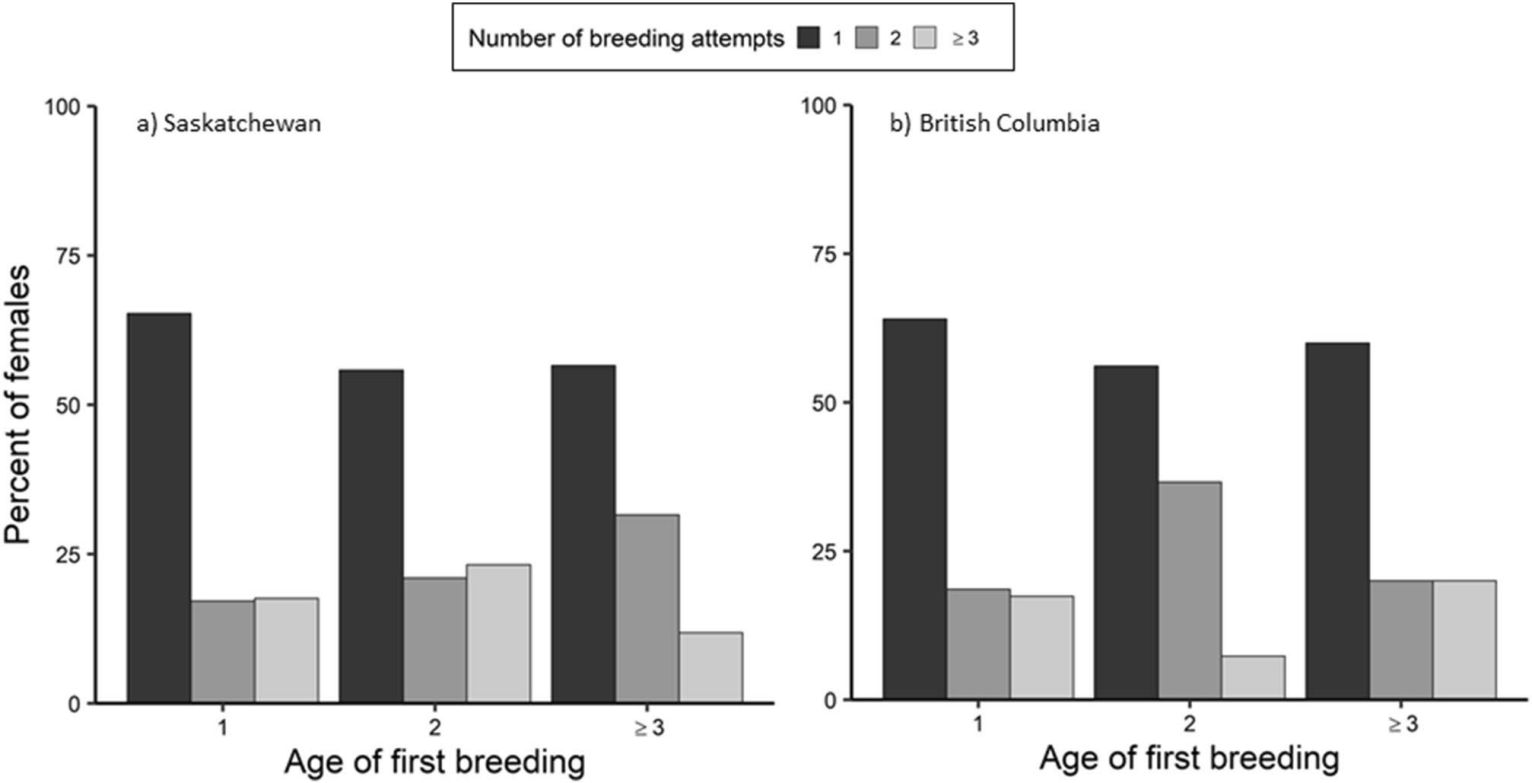
Percent of known-age female tree swallows in (**a**) Saskatchewan (1990–2015; *n* = 496) and (**b**) British Columbia (2001–2015; *n* = 466) making 1, 2, or ≥ 3 lifetime total breeding attempts for each age of first breeding (1, 2, or ≥ 3 years old).

Short-lived species generally breed only once in their lifetime^62^, which is consistent with our finding of female tree swallows making only a single breeding attempt regardless of their age of first breeding (Fig. 3). While environmental factors, such as pond abundance and winter conditions can influence female survival^64^, we examined whether female quality influenced number of breeding attempts (Supplemental Analysis 1). Similar to LRS, we found no strong effects of female body condition or age of first breeding on number of breeding attempts, but females that were relatively earlier season breeders made more breeding attempts (Table S1 and Supporting Supplementary Text). Nevertheless, given the importance of survival to LRS, we encourage further studies to conduct formal survival analyses incorporating measurements of individual quality and environmental factors across the annual cycle. We also recognize the possibility that we may have detected only a single breeding attempt for most females because they dispersed, especially at the BC site where fledgling success was low^11^, or bred elsewhere or in natural cavities before acquiring a nest box at the SK site given nest box occupancy was high^67^. Breeding site dispersal for adult tree swallows is low^68^, but fewer yearling females were detected breeding at the SK site compared to BC. We cannot fully account for permanent dispersal of juveniles^69^ or the fact that female tree swallows could breed as yearlings elsewhere, leading to underestimates of the number of females making more than one breeding attempt. Therefore, while our results for tree swallows are consistent with the general patterns of LRS for short-lived species^9,10^, we report ‘apparent’ estimates of LRS and urge other studies to do so as well.

Our results suggest that climate change projections of increased frequency of extreme climate events may generate large, and perhaps distinct, effects on apparent LRS of species across their range. In the prairies where current warmer, wetter conditions associated with climate change appear to be benefiting some species^70^, climate scenarios are forecasting more frequent and severe drought conditions and widespread drying of ponds^71,72^, which our results suggest could negatively affect the fitness of terrestrial species. Such effects will not only occur via habitat loss, but also through the loss of aquatic food resources for a variety of species. In the interior of BC, predictions of increased precipitation^73^ combined with unpredictable cold weather events experienced by early-season breeders may create poor breeding conditions. Precipitation reduces the availability of aerial insect food resources, with negative consequences on offspring quality and population growth^74^. Although the effects we detected were weak, increased frequency of inclement weather has the potential to exacerbate these effects in the future. In contrast, increased precipitation leading to wetter conditions and earlier breeding combined with warmer spring temperatures have the potential to create more favorable conditions during breeding.

By evaluating the effects of individual characteristics and weather fluctuations and climate extremes using a lifetime perspective in a short-lived avian species, we discovered that most individuals bred only once and environmental conditions can generate large variation in lifetime fitness. Given that changes in climate are affecting local environments producing wetter or drier, and possibly warmer conditions, we predict that geographic differences in LRS may be a strong determinant of population change caused by climate variation.

## Materials and methods

### Study species

Tree swallows are migratory, aerial insectivores that breed throughout the central and northern parts of North America^30^. In most of its range, this species produces a single brood per season with clutch sizes ranging from 1–9 eggs, although clutches containing 4–7 eggs are most common^30^. Extra-pair paternity in this species is high, with up to 85% of broods containing at least one extra-pair offspring^75^, but egg dumping by other females is rare^76^. The average lifespan of tree swallows is approximately 2.7 years^30^. Female apparent survival probability is approximately 50%^64^, while juvenile apparent survival probability is 6% at the SK site and 5% in BC^11^. Female tree swallows show high breeding site fidelity^68^ and do not generally disperse after a season with poor breeding success^77^. Female tree swallows intensively compete for nest sites^78^, and because nest sites may be limited, females in their second-year (SY) of life (i.e., their first breeding season) are sometimes competitively excluded from breeding in their first season^79^.

### Study areas and general field methods

Tree swallows were studied in Canada at a Saskatchewan (SK) site, 1990–2017, and at a site in British Columbia (BC), 2001–2017. The SK site is located in the prairie pothole region, at the St. Denis National Research Area, 40 km east of Saskatoon (52^o^N, 106^o^W); the area contains > 100 wetland basins of varying permanency (when flooded, basins contain “ponds”), as well as grassland, cultivated land, and groves of aspen (*Populus tremuloides*)^80^. The BC site, located within 30 km of Prince George (53° N, 123° W), is in the sub-boreal forest and has cooler temperatures, and higher spring and summer precipitation (Table S1). The BC site consists of hayfields intermixed with stands of deciduous trees and few small permanent wetlands^81^. At both sites, wooden nest boxes are mounted on metal or wooden posts spaced approximately 20–30 m apart. Further details regarding these sites are described in Shutler and Clark^77^, Dawson et al*.*^82^, Bitton and Dawson^83^, and Harriman et al*.*^50^.

Reproductive data from breeding tree swallows were collected from May to August. Nest boxes were visited regularly to record clutch initiation date (i.e., day the first egg was laid, where 1 January = 1), clutch size, hatching date, and number of nestlings hatching. Most adult tree swallows were captured within a few days after the last egg in a nest hatched; in a few instances, females were captured prior to the hatching of eggs^64^. Captured birds were identified as female or male by the presence of a brood patch or cloacal protuberance, respectively, and unbanded adults were banded with an individually numbered aluminum leg band. Because of consistent capture and banding efforts for tree swallows at our sites over the study period, nearly every breeding adult and all nestling tree swallows were banded, and recapture probabilities were high^11^. Upon capture, most adults were weighed using a spring balance (± 0.5 g) and wing length was measured using a ruler (± 0.5 mm). Nestling were banded either when they were 12 or 16 days old. Nests were visited 20 days after hatching to record the number of nestlings that successfully fledged.

### Path model hypotheses and data analysis

Data on annual reproductive success for known-age adult female tree swallows breeding in SK (1990–2015) and BC (2001–2015) were summarized over all breeding attempts made by each female. We included females that bred up to 2015 and detected recruits up to 2017. Analyses were based on 496 (SK) and 466 (BC) known-age adult female tree swallows with complete information for all explanatory variables (see below). Exact age was known with certainty for females banded as nestlings or birds identified as first-year breeders (i.e., SY) according to their dorsal plumage color^84^. Females involved in studies that could influence survival and hence LRS (e.g., those deployed with geolocators^85^) were excluded. Data for males were not analyzed; unlike females, estimates of apparent LRS would be unreliable given frequent extra-pair paternity^75^.

We included nine explanatory variables for the breeding lifespan of each female that we hypothesized would influence LRS (Table 3). We expected that the age of a female’s first breeding attempt, ‘Age of First Breeding’, would lead to higher LRS for females that begin breeding as yearlings because they would make more breeding attempts than females that delayed breeding until two years of age or older^86^ (Table 3). We also expected that females making more breeding attempts in their lifetime, ‘Total Breeding Attempts’, would produce more recruits directly^20^ or indirectly because they laid more eggs, ‘Total Eggs’, and produced more fledgling, ‘Total Fledglings’, in their lifetime^37^ (Table 3). Minimum clutch size or fledged offspring were used in rare instances when exact values were unknown (*n* = 10 [SK] and *n* = 3 [BC]). In a few cases (*n* = 9 for SK, *n* = 6 for BC), we excluded a breeding attempt if that attempt resulted in no fledged offspring (e.g., human-caused nest failure). Few females re-nested after brood failure (*n* = 5 at each site) and we only counted such cases as a single breeding attempt, but we used the clutch initiation date and clutch size from the first breeding attempt and number of fledged offspring from the re-nesting attempt, following Murphy^37^, when averaging or summing values over all breeding attempts.

**Table 3:** Definitions of variables and their hypothesized relationship to lifetime reproductive success of known-age adult female tree swallows.

Variable	Definition	Predicted relationship to LRS	Hypothesis	Reference
Lifetime reproductive success	Number of recruited offspring produced by adult females summed over all breeding attempts			
Total fledglings	Number of fledged offspring produced by adult females summed over all breeding attempts	+	Females fledging more offspring produce more recruits	^27,37^
Total eggs	Number of eggs laid by adult females summed over all breeding attempts	+	Females laying more eggs fledge more offspring and produce more recruits	^27,37^
Total breeding attempts	Number of breeding attempts (first clutches) made by adult females summed over their lifetime	+	Females making more breeding attempts produce more recruits	^20,27,37^
Relative initiation date	Egg laying date of adult females relative to the annual 5% clutch initiation date averaged over all breeding attempts	–	Later season breeding females lay fewer eggs and produce fewer recruits	^18,42,50^
Relative condition	Body mass of adult females controlling for structural size, age category (SY/ASY^a^), year, and the number of days between egg laying and capture date averaged over all breeding attempts	+	Females with higher body condition are high-quality or have access to greater resources, and produce more fledglings	^29,87,88^
Age of first breeding	Age of a female’s first detected breeding attempt	+	Females that begin breeding at 1-year-old make more breeding attempts and produce more recruits	^86^
Relative ponds	Total number of ponds (wetland basins containing water) in May summed over all breeding attempts and mean-centered to the pond value for the number of breeding attempts made (i.e., 1, 2, 3, and ≥ 4)	+	Females breeding when there are more ponds, reflecting higher food quantity or quality, produce more fledglings	^32,33^
Average PDSI	The mean Palmer Drought Severity Index for June and July (proxy for local moisture conditions) averaged over all breeding attempts	+	Females breeding when moisture conditions are greater, reflecting higher food quantity or quality, produce more fledglings	^32,33^
Relative severity	Number of cold snaps (i.e., days with maximum temperature below 18.5 °C) spanning two to seven days, multiplied by the number of days nestlings were in the nest and weighted for severity (1 for those spanning 2 days or 2 for those longer than 3 days). These values were summed over all breeding attempts and mean-centered to the severity value for the number of breeding attempts made (i.e., 1, 2, 3, and ≥ 4)	–	Females encountering cold weather when nestlings are 4–12 days old produce fewer fledglings	^5,31^

See “Materials and methods” section for further details.

^a^SY = second-year and ASY = after-second-year. Refer to “Materials and methods” for more details.

Female tree swallows that breed earlier in the season are higher quality individuals^18^ so we anticipated that females breeding relatively early in the season throughout their lifetime would have higher LRS because they lay more eggs^42^ and raise nestlings when insect food resources are more plentiful^50^ (Table 3). This measure of ‘Relative Initiation Date’ is the average standardized clutch initiation date for all breeding attempts, calculated by subtracting the annual 5% clutch initiation date for all nests from each individual female’s initiation date following Clark et al*.*^89^. Initiation date was standardized separately for after-second-year (ASY) and SY females because SY females generally breed later than ASY females^90^.

Early season breeding females are heavier^29^, better able to acquire insect food resources^87,88^ and can invest in self-maintenance while breeding^91^. Therefore, we expected females that were relatively early season breeders during their life to be in higher body condition, ‘Relative Condition’, when captured while rearing nestlings and make more lifetime breeding attempts (Table 3). Relative Condition was the residual from a linear model that included body mass as the response variable and female age category (SY or ASY), wing length, year, and the number of days from when females initiated their clutch to being captured as explanatory variables. Residuals were averaged over all breeding attempts, except for 27 (SK) and 41 (BC) females where mass or wing measurements were missing and average values were calculated with the available data. Time of capture may influence the body mass of females^48^. Although females at the Saskatchewan site were mainly captured in the morning, exact capture times are unknown, so we explored how capture time influenced body mass for 451 of 466 females with data for capture time at the British Columbia site. While the body mass of known-age adult females was positively related to time of capture (standardized estimate (SE), β = 0.24 ± 0.05), body condition residuals for these females computed with and without capture time were nearly identical (linear regression estimate (SE), β = 1.00 ± 0.01).

Cold, inclement weather can lower insect availability^31^ and result in nestling mortality^5^; therefore, we hypothesized that the lifetime relative number of cold days experienced by females when their nestlings were 4–12 days old, ‘Relative Severity’, would have negative effects on lifetime fledgling production (Table 3). Relative Severity was calculated only for BC because 67% of females experienced severe cold weather during breeding, whereas 65% of females in SK experienced no cold snaps in their lifetime. Cold days were calculated as the number of cold snaps, i.e., days when maximum temperature was below 18.5 °C, identified by Winkler et al*.*^31^ as the critical temperature for insect flight, that spanned between two to seven days, multiplied by the number of days offspring were in the nest. We did not use cold snaps consisting of only one day because a daily fluctuation below 18.5 °C during a period of favorable weather is unlikely to affect nestling survival. Additionally, since longer cold snaps may be more severe than shorter ones, those spanning three or more days were weighted by 2, whereas cold snaps of 2 days were weighted by 1. Relative severity is the number of cold days, summed over all of a female’s breeding attempts, and because females making more breeding attempts have higher cumulative number of cold days, we centered to the mean cold severity value for the number of breeding attempts made (i.e., 1, 2, 3, and ≥ 4). Daily maximum temperature data were retrieved from Environment and Climate Change Canada (https://climate.weather.gc.ca/index_e.html).

Aerial insects, the main food of swallows^30^, can influence the timing of clutch initiation^92^ and investment in reproduction^88^, and because insect abundance is greater in years with abundant ponds^32^ we expected lifetime abundance of ponds, ‘Relative Ponds’, experienced by females to influence fledgling production (Table 3). Relative ponds is the number of wetland basins containing water in May at the SK site (Fig. S2), summed over all of a female’s breeding attempts. Although higher May pond counts influence survival of adult females at Saskatchewan^64^, we separated total breeding attempts from pond abundance by mean-centering the cumulative pond value for the number of breeding attempts made (i.e., 1, 2, 3, and ≥ 4) because females making more total breeding attempts have a higher cumulative total pond count. Given that wetlands are generally permanent in BC, we used the average Palmer Drought Severity Index (PDSI) for June and July, which has been shown previously to provide a suitable measure of local moisture conditions at our study sites^33^. For instance, at the SK site, PDSI is positively correlated both with number of ponds (this study: *r* = 0.59, *p* = 0.001, *n* = 26) and their depth^33^ in May. Site-specific PDSI values were obtained from Agriculture and Agri-Food Canada.

We performed confirmatory path analysis^26^ using the package PIECEWISESEM^93^ in R (version 3.6.1)^94^ to test causal hypotheses explaining how individual traits and environmental factors directly and indirectly affect apparent LRS of female tree swallows. Extreme values of some variables were truncated^89^ and log-transformed when they improved the fit of data to linear models testing claims of conditional independence between explanatory variables (see below). We simultaneously performed analyses using SAS (PROC CALIS)^95^ and the standardized residuals and goodness of fit tests additionally confirmed that final models provided a very good fit to the data.

Our hypothesized path model was evaluated using Shipley’s goodness of fit test, the Fisher’s C statistic, provided by the PIECEWISESEM package^93^. Path models that fail the goodness of fit test have a significant *p*-value, indicating that there are associations between variables (i.e., paths) missing from the model^26^. These missing paths are identified by performing simultaneous tests of conditional independence between variables not included in the path model and significant *p*-values indicate missing paths^26,93^, which were then added to the path model. Non-significant paths were removed from the model until only significant pathways remained^58^. A final model with a non-significant *p*-value indicated good fit to the data^26^. Standardized path coefficients for direct effects between variables were extracted from the final path model; total effects of variables were calculated by multiplying standardized path coefficients for each indirect path, and then summing all indirect and direct pathways^20^.

For our path analyses, Total Fledglings and apparent LRS (= total recruits) reflect offspring raised in the nest of a female because 28% of females at SK and 21% of females at BC were involved in experiments where eggs or nestlings were swapped, added or removed from nests^50,77^. Our main results and conclusions were unchanged when we limited analyses to fledglings and recruits genetically related to the female (*n* = 449 [SK] and *n* = 448 [BC]).

We tested for correlations in lifetime number of breeding attempts, eggs laid, fledglings produced, and relative initiation date between 26 sister (*n* = 16 SK, *n* = 10 BC) and 140 mother-daughter (*n* = 71 SK, *n* = 69 BC) pairings. Variables were standardized (mean = 0, standard deviation = 1) separately by site, and data were then pooled prior to analysis using Spearman’s correlations. We were unable to test whether apparent LRS or age of first breeding were positively related between sisters and mother and their daughters because there was limited variation in these variables due to the majority of females producing no recruits or breeding at 1 or 2 years old.

### Animal handling and ethics

All handling and research on tree swallows were approved by institutional animal care committees, the University of Saskatchewan and University of Northern BC, and carried out in accordance with the Canadian Council on Animal Care guidelines.

## Supplementary information

Supplementary Information.

## Data Availability

Data available upon reasonable request from L.L.B. and R.G.C.
