# Supplementary material for: The relative contribution of individual quality and changing climate as drivers of lifetime reproductive success in a short-lived avian species
Source: Sci Rep. 2020 Nov 13;10:19766. doi: 10.1038/s41598-020-75557-w (PMC7666198; doi:10.1038/s41598-020-75557-w)
Supplement: Supplementary file 1 — Supplementary Information. [file 41598_2020_75557_MOESM1_ESM.pdf]

Supplementary Information

Individual quality and changing climate: Disentangling drivers of lifetime reproductive success in a short-lived avian species

Lisha L. Berzins[1,*], Russell D. Dawson[2], Christy A. Morrissey[1,3], Robert G. Clark[1,4]

[1] University of Saskatchewan, Department of Biology, Saskatoon, SK, S7N 5E2, Canada
[2] University of Northern British Columbia, Ecosystem Science and Management Program, Prince George, BC, V2N 4Z9, Canada
[3] University of Saskatchewan, School of Environment and Sustainability, Saskatoon, SK, S7N 5C8, Canada
[4] Environment and Climate Change Canada, Saskatoon, S7N 0X4, Canada
*lisha.berzins@usask.ca

Supplemental Analysis 1 - Number of breeding attempts and characteristics of individual females

Previously, we showed that apparent survival rates of adult female tree swallows were related to spring weather and local pond conditions in Saskatchewan (SK) and, possibly, winter climate conditions at SK and Prince George, British Columbia (BC)[1]; thus, these variables must influence number of breeding attempts because female tree swallows are single-brooded at our study sites. However, Clark et al.[1] did not relate apparent survival to measurements of individual quality, like timing of breeding or body condition. Early-breeding females with higher body condition may survive better, increasing their number of lifetime breeding attempts. Females that are able to breed at one-year-old may also be higher-quality individuals and, in relatively short-lived songbirds, early start of breeding (i.e., at 1 year old) is expected to be a more successful strategy.

Due to the apparent importance of number of breeding attempts for lifetime reproductive success we investigated whether number of attempts made by known-age adult female tree swallows was related to indices of individual quality. We performed multinominal regression as implemented with PROC GENMOD in SAS 9.4[2]. We analyzed all data in a common modelling framework because explanatory variables were measured in the same manner on both sites. Number of breeding attempts (range: 1-4) was related to relative clutch initiation date (standardized by site), body condition index, age at first breeding (1 year old, or 2 and older), site (SK,BC), and 2-way interactions between these main effects. (A random effects structure [PROC GLIMMIX, with random effects of year] produced very similar results and conclusions and are not reported.) Models were ranked by Akaike's Information Criterion (AIC).

The most parsimonious results involved nonlinear relationships between number of breeding attempts and clutch initiation date that depended on age at first breeding (Table S1). In general, late-nesting females made fewer breeding attempts. However, among females that bred at 1 year old (n = 659), the earliest and especially the later-nesting females made fewer attempts (β_{zCID} = 0.554, 95% Wald confidence interval [CI] = 0.287, 0.821; $\beta_{zCID}{}^2$ = -0.528, CI = -0.732, -0.333). Among females detected nesting for the first time at 2 years or older (n = 303), late-nesting females made fewer lifetime breeding attempts (β_{zCID} = -0.182, CI = 0.225, -0.623; $\beta_{zCID}{}^2$ = -0.434, CI = -0.865, -0.003). These patterns were consistent at both sites. No other models were highly competitive against the best-approximating model (Table S1; ΔAIC > 4). Site effects were negligible, and no two-way interactions were found between any of the other main effects.

Overall, late-nesting females had fewer lifetime breeding attempts. We also conclude that number of breeding attempts is positively related to early-mid season breeding by females that start breeding at one year old, and earlier breeding among females detected at older ages. These seasonal patterns could reflect higher individual quality or possibly some aspect of environmental quality. Early season environmental conditions may present greater challenges

for younger females. Formal survival analyses could confirm and clarify these relationships, and evaluate the relative importance of other covariates of interest.

Supplemental Table S1. Model selection results for multinomial models to account for number of lifetime breeding attempts by known-age, adult female tree swallows from St. Denis, Saskatchewan (SK; *n* = 496), and Prince George, British Columbia (BC; *n* = 466). Shown are model structures, Akaike's Information Criterion (AIC), change in AIC between the top-ranked and lower-ranked models (ΔAIC), model weight (*w*), number of parameters (*k*) and -2 log likelihood (-2LL).

Model	AIC	delta-AIC	*k*	-2 LL
zCID, zCID^2, AgeFB, zCID*AgeFB	1961.16	0.00	7	1947.16
zCID CID^2	1967.78	6.62	5	1957.78
zCID, zCID^2, AgeFB	1968.42	7.26	6	1956.42
zCID, zCID^2, AgeFB, COND, Site, zCID*Site, zCID*AgeFB, zCID*Cond, AgeFB*Site AgeFB*Cond, Cond*Site	1971.28	10.12	14	1943.28
zCID	2012.17	51.01	4	2004.17
Intercept	2020.67	59.50	3	2014.67

[a] zCID is the z-score for relative clutch initiation date, standardized by site; zCID^2 is the quadratic term for zCID; AgeFB is age at first detected breeding (1 or ≥2 years old); Cond is body condition index; Site refers to study site location, BC or SK.

[b] All models contained three intercepts, representing 3 of 4 levels of breeding attempts.

Supplemental Table S2 Descriptive statistics (mean ± standard error, and range) of individual traits and environmental factors for known-age adult female tree swallows breeding in Saskatchewan (1990-2015) and British Columbia (2001-2015). Refer to Methods for details about calculations of individual and environmental factors.

	Saskatchewan			British Columbia		
	**Mean ± SE**	**Range**	***N***	**Mean ± SE**	**Range**	***N***
Clutch size	6.2 ± 0.04	1 – 10	828[c]	5.8 ± 0.03	2 – 9	696[c]
Clutch initiation date	28 May ± 0.3	15 May - 13 July	828	31 May ± 0.3	13 May – 9 July	696
Number of fledged offspring	5.2 ± 0.2	0 – 10	828	3.6 ± 0.1	0 – 9	696
Adult female body mass (g)	21.6 ± 0.1	16.5 – 27.5	828	19.6 ± 0.1	16.3 – 23.8	696
Cold severity index[a]	1.94 ± 0.2	0 – 22	496	6.24 ± 0.4	0 – 40	466
Maximum June temperature	22.1 ± 0.3	19.4 – 25.0	26[d]	20.5 ± 0.4	18.2 – 22.9	15[d]
PDSI[b]	0.6 ± 0.4	-3.8 – 6.1	26	1.1 ± 0.2	-0.3 – 3.3	15
Ponds	64.8 ± 6.8	2 – 107	26	-	-	-

[a]Cold severity index -number of cold days (below 18.5^{o}C) experienced by females when their offspring were 4-12 days old (see Methods for description of calculation of cold severity).

[b]PDSI -Mean Palmer drought severity index for June and July. Negative values signify drier conditions.

[c]Number of females at SK and BC.

[d]Number of years at SK and BC.

Supplemental Figure S1 Relationship between relative clutch initiation date and relative number of recruited offspring produced by known-age adult female tree swallows in Saskatchewan (1990-2015; *n* = 496) and British Columbia (2001-2015; *n* = 466). Relative number of recruits is the proportion of nestlings that recruited in relation to the total number of nestlings produced. Numbers above symbols represent the number of females in each interval of relative clutch initiation date (see Methods for description about calculation of Relative Initiation Date).

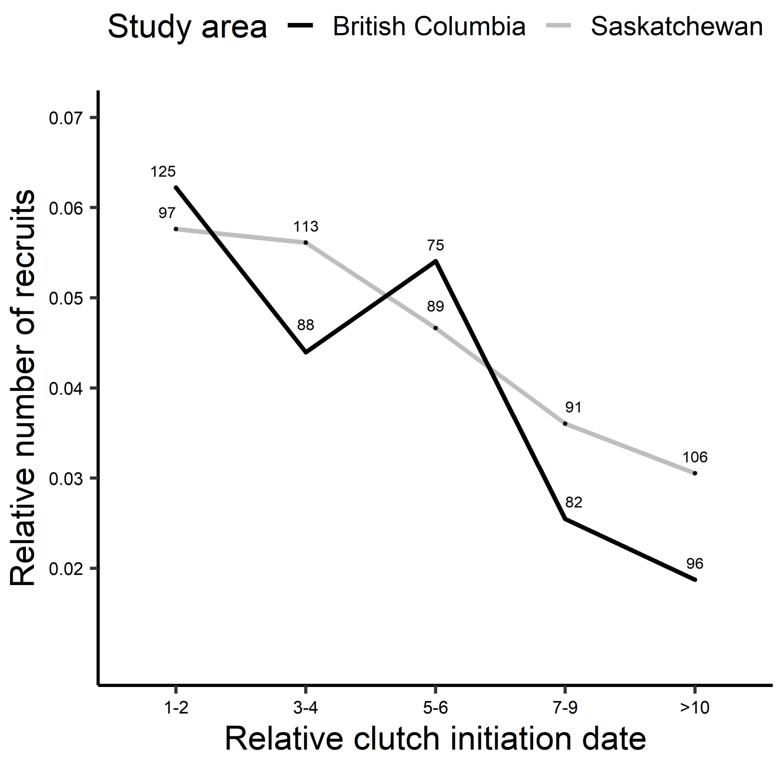

Supplemental Figure S2 Number of ponds at the Saskatchewan site from 1990-2015. 'Ponds' are wetland basins containing water when counted in early-mid May.

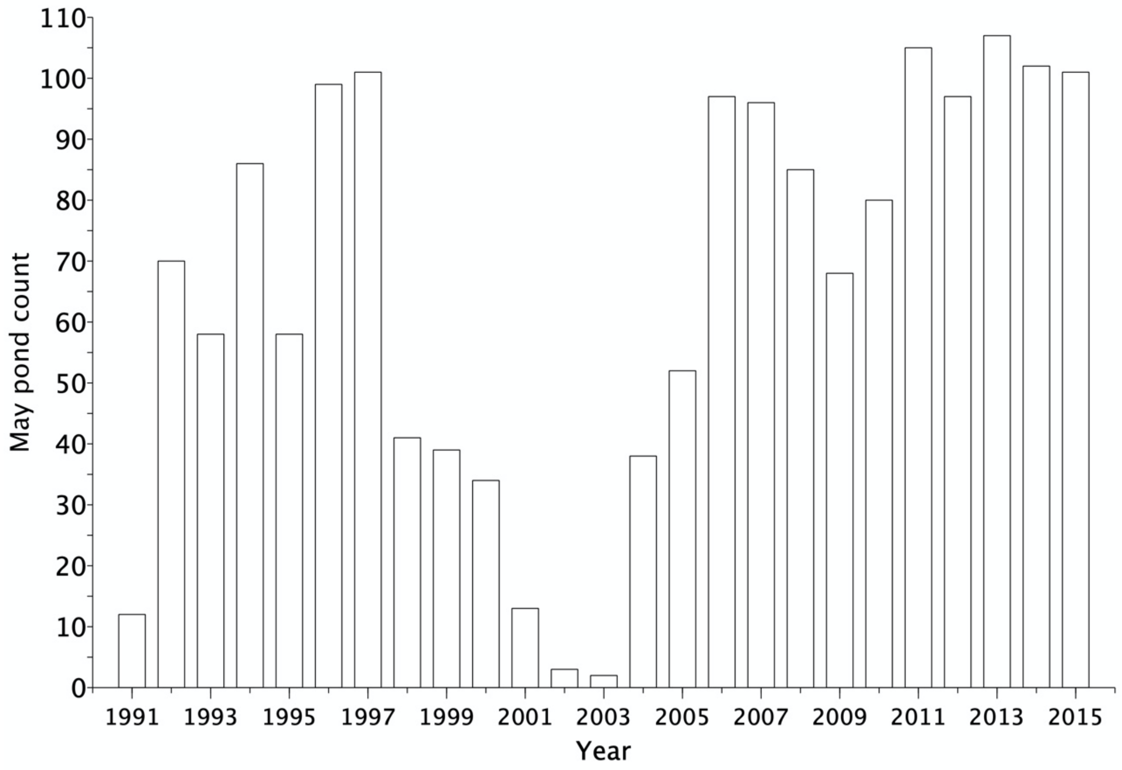

References

1. Clark, R. G. *et al.* Geographic variation and environmental correlates of apparent survival rates in adult tree swallows *Tachycineta bicolor*. *J. Avian Biol.* **49**, 012514 (2018).

2. SAS Institute Incorporated. *SAS (Data Analysis Software System), Version 9.4.* (SAS Institute Incorporated., 2016).
